# Simvastatin is associated with superior lipid and glycaemic control to atorvastatin and reduced levels of incident Type 2 diabetes, in men and women, in the UK Biobank

**DOI:** 10.1002/edm2.326

**Published:** 2022-03-04

**Authors:** Andrew R. English, Bodhayan Prasad, Declan H. McGuigan, Geraldine Horigan, Maurice O’Kane, Anthony J. Bjourson, Priyank Shukla, Catriona Kelly, Paula L. McClean

**Affiliations:** ^1^ Northern Ireland Centre for Stratified Medicine School of Biomedical Sciences C‐TRIC Altnagelvin Hospital Ulster University Derry~Londonderry UK; ^2^ Clinical Chemistry Laboratory Altnagelvin Hospital Derry~Londonderry UK; ^3^ Centre for Personalised Medicine: Clinical Decision Making and Patient Safety C‐TRIC Altnagelvin Hospital Londonderry UK

**Keywords:** gender differences, glycaemic control, HbA1c, lipid control, statin, type 2 diabetes, UK Biobank

## Abstract

**Introduction:**

Cardiovascular disease (CVD) is the leading cause of mortality in people with Type 2 diabetes mellitus (T2DM). Statins reduce low‐density lipoproteins and positively affect CVD outcomes. Statin type and dose have differential effects on glycaemia and risk of incident T2DM; however, the impact of gender, and of individual drugs within the statin class, remains unclear.

**Aim:**

To compare effects of simvastatin and atorvastatin on lipid and glycaemic control in men and women with and without T2DM, and their association with incident T2DM.

**Methods:**

The effect of simvastatin and atorvastatin on lipid and glycaemic control was assessed in the T2DM DiaStrat cohort. Prescribed medications, gender, age, BMI, diabetes duration, blood lipid profile and HbA1c were extracted from Electronic Care Record, and compared in men and women prescribed simvastatin and atorvastatin. Analyses were replicated in the UKBiobank in those with and without T2DM. The association of simvastatin and atorvastatin with incident T2DM was also investigated in the UKBiobank. Cohorts where matched for age, BMI and diabetes duration in men and women, in the UKBioBank analysis, where possible.

**Results:**

Simvastatin was associated with better LDL (1.6 ± 0.6 vs 2.1 ± 0.9 mmol/L, *p* < .01) and total cholesterol (3.6 ± 0.7 vs 4.2 ± 1.0 mmol/L, *p* < .05), and glycaemic control (62 ± 17 vs 67 ± 19 mmol/mol, *p* < .059) than atorvastatin specifically in women in the DiaStrat cohort. In the UKBiobank, both men and women prescribed simvastatin had better LDL (Women: 2.6 ± 0.6 vs 2.6 ± 0.7 mmol/L, *p* < .05; Men: 2.4 ± 0.6 vs 2.4 ± 0.6, *p* < .01) and glycaemic control (Women:54 ± 14 vs 56 ± 15mmol/mol, *p* < .05; Men, 54 ± 14 vs 55 ± 15 mmol/mol, *p* < .01) than those prescribed atorvastatin. Simvastatin was also associated with reduced risk of incident T2DM in both men and women (*p* < .0001) in the UKBiobank.

**Conclusions:**

Simvastatin is associated with superior lipid and glycaemic control to atorvastatin in those with and without T2DM, and with fewer incident T2DM cases. Given the importance of lipid and glycaemic control in preventing secondary complications of T2DM, these findings may help inform prescribing practices.

## Graphical abstract

Statins have secondary effects on glycaemia and diabetes onset risk; it is unknown if this differs between men and women. In DiaStrat, we found women, not men, have superior lipid and glycaemic control in response to simvastatin compared with atorvastatin. Validation analysis using the UK Biobank cohort indicated that men and women with T2DM achieve superior lipid and glycaemic control with simvastatin than atorvastatin and reduced incident T2DM. These findings may inform prescribing practices, with respect to statins, in those at‐risk of and with existing T2DM.

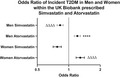

**Novelty Statement**

- Statins have secondary effects on glycaemia and diabetes onset risk; it is unknown if this differs between men and women.
- In DiaStrat, we found women, not men, have superior lipid and glycaemic control in response to simvastatin compared with atorvastatin.
- Validation analysis using the UK Biobank cohort indicated that men and women with T2DM achieve superior lipid and glycaemic control with simvastatin than atorvastatin.
- Simvastatin prescription reduced the risk of incident T2DM in the UK Biobank compared with atorvastatin in men and women.
- These findings may inform prescribing practices, with respect to statins, in those at‐risk of and with existing T2DM.

## 1 INTRODUCTION

Type 2 diabetes mellitus (T2DM) and co‐morbid CVD, lipid dysregulation and hypertension, are driven by common aetiologies of obesity and sedentary behaviour. Controlling diseases of the circulatory system are a key objective in the management of T2DM, in an attempt to prevent the increased morbidity and mortality associated with CVD in this population. In addition to lifestyle and dietary improvements, most clinical guidelines recommend statin use in T2DM for CVD prophylaxis.^1^ The National Institute for Health and Clinical Excellence (NICE)^2^ recommends that people over the age of 40 years with T2DM are prescribed statins. Statins, 3‐hydroxy‐3‐methylglutaryl coenzyme A (HMG‐CoA) reductase inhibitors, are an effective cholesterol‐lowering drug class that reduce low‐density lipoprotein (LDL) cholesterol levels, and have antioxidant and cardioprotective properties.^3^ However, recent evidence indicates that statin therapy is associated with increased drug interactions, poor diabetes outcomes and potential worsening of glycaemic control in those on high‐dose statin therapy.^4^ This is also supported in a retrospective cohort study of 12,725 participants from the health improvement network (THIN) where concurrent statin use was associated with higher HbA1c levels, after initiation of insulin, throughout a 3‐year follow‐up, relative to those not in receipt of statin therapy.^5^

As a drug class, statins increase the risk of developing T2DM by 10%–12%.^6^ However, this effect appears to be heavily influenced by statin type and dose. A network meta‐analysis of 163,039 participants revealed high‐dose atorvastatin increased the odds of developing diabetes compared with low‐dose atorvastatin.^7^ In addition to increasing the risk of new‐onset T2DM, statins have also been shown to alter the function of insulin‐secreting beta cells and to increase insulin resistance, suggesting a potential diabetogenic effect for the drug class.^6^ Disturbances in insulin and glucose homeostasis significantly increase the potential for major adverse cardiac events.^8^

More work is required to differentiate statins with respect to their effect on blood lipids and glycaemic control, and little research has been done on how gender can influence response. The aims of this study were as follows: (1) To characterize the DiaStrat T2DM cohort in Northern Ireland (NI), in terms of comorbidities influencing glycaemic control and gender differences in lipid and glycaemic control in response to the most widely prescribed statins, simvastatin and atorvastatin. (2) To expand the research question to the UK Biobank T2DM population to assess the generalizability of the findings (as the UK Biobank includes participants from England, Scotland and Wales (but not NI)). (3) To utilize the UK Biobank dataset to investigate lipid and glycaemic control associated with statin prescription in those without diabetes and (4) To further investigate the association with incident diabetes.

## 2 PARTICIPANTS AND METHODS

### 2.1 The DiaStrat cohort

The stratified medicine optimizing treatment for diabetes (DiaStrat) study is a pilot observational study. A total of 500 adults aged between 18 and 80 years, with clinically diagnosed T2DM were enrolled in the study from diabetes clinics in the Western Health and Social Care Trust (WHSCT) in Northern Ireland between May 2015 and March 2017, informed consent was obtained from the patient. Those >80 years old or with other forms of diabetes were excluded. This was based on the average age of individuals treated for diabetes in secondary care clinics in our local trust area. Many older patients are treated in a primary care setting. Relevant clinical information for all participants was obtained from the Northern Ireland Electronic Care Record (NIECR; Orion health) at the date of recruitment, for this cross‐sectional study. Data collected included gender, date of birth (DOB), age at diagnosis, biochemical lab values, all prescription data and recorded comorbidities. Data were not available for all variables, and the revized ‘n’ is indicated in Tables/Figures. Three hundred and seventy four participants provided a blood sample, from which plasma was extracted, permitting analysis of c‐peptide.

The present study focused on comorbid endocrine disorders in the DiaStrat cohort, which were primarily associated with lipid abnormalities, as this comorbidity was associated with inferior glycaemic control (Table 3). A diagnosed lipid abnormality was defined where a participant had a diagnosis of hyperlipidaemia recorded within their ECR by a clinician. Within this group, we assessed lipid regulating medications (428 total). Due to the prevalence of atorvastatin and simvastatin prescription (389, 91%), analyses focused on the presence or absence of both drugs and assessed differences in glycaemic and lipid control in men and women.

### 2.2 UK biobank analyses

The UK Biobank (ukbiobank.ac.uk) has approximately 500,000 participants, aged 40–69 years, recruited between 2006 and 2010, from the general population of the United Kingdom.^9^

We replicated part of our DiaStrat analyses using the UK Biobank by extracting data for participants with a confirmed diagnosis of T2DM (using date ICD‐10 code E11 first reported, Field‐ID 130708), prescribed either simvastatin or atorvastatin (*n* = 16,257). Age, duration of diabetes, BMI, blood lipids and HbA1c were also extracted. We further divided participants into those diagnosed with T2DM before recruitment (simvastatin: *n* = 5496; 3534 men and 1962 women; atorvastatin: *n* = 2227; 1431 men and 796 women, Table 5) and participants were diagnosed with T2DM after recruitment, (incident T2DM; simvastatin: *n* = 8534; 5454 men and 3080 women; atorvastatin: *n* = 2516; 1617 men and 899 women, Table 7). For incidence T2DM, we report baseline characteristics of those who developed T2DM after baseline, as follow‐up biochemical analyses were not available for such participants. The effect of simvastatin (*n* = 42,816; 25,593 men and 17,223 women) and atorvastatin (*n* = 10,241; 6310 men and 3931 women) prescription on blood lipids and HbA1c was also assessed in the absence of diabetes (ICD‐10 codes E10 and E11, Field‐ID 41270, Table 6).

### 2.3 C‐peptide enzyme‐linked immunosorbent assays (ELISA)

Plasma c‐peptide was measured for *n* = 374 participants of the DiaStrat cohort using human Alpco c‐peptide ELISA kit (Alpco; Cat no. 80‐CPTHU‐E01.1, E10), according to the manufacturer's instructions. No c‐peptide data were available for UK Biobank participants.

### 2.4 Blood lipid measurement

High‐density lipoprotein (HDL) cholesterol, total cholesterol and triglycerides where measured via direct laboratory assay (Cobas C‐701 analyser). Low‐density lipoprotein (LDL) cholesterol was calculated using the Friedewald formula.

### 2.5 Statistical analysis

Biochemical changes in the DiaStrat analysis were determined in SPSS version 25 using two‐way ANOVA with Bonferroni post hoc analysis. Analysis of the UK Biobank dataset was carried out in the open source software, R (https://www.R‐project.org/). The UK Biobank fileset was loaded in the R environment using ‘ukbtools’ package (https://kenhanscombe.github.io/ukbtools/). Means, standard deviations, two‐sample T‐tests and one‐way ANOVAs were calculated within the base R package. Significance threshold was set at *p* < .05 for all analysis.

**FIGURE 1:**
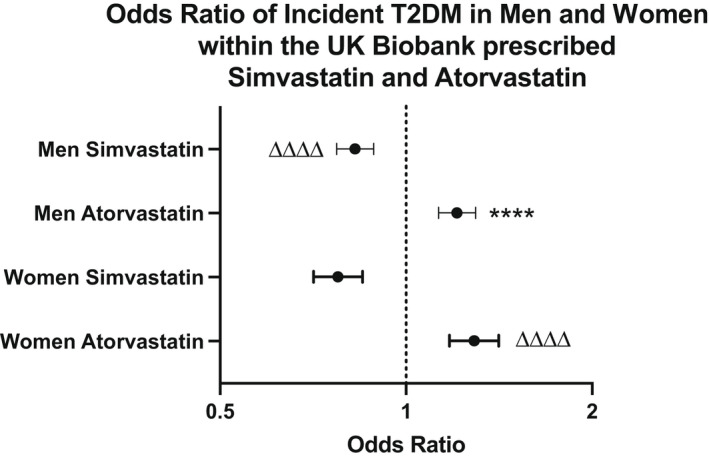
Odds ratio of incident T2DM in men and women from the UK Biobank, without T2DM at baseline, prescribed simvastatin or atorvastatin. Individuals prescribed simvastatin and atorvastatin, without a diagnosis of T2DM, were identified at baseline within the UK Biobank cohort. Incident T2DM was noted when an individual received a T2DM diagnosis after the UK Biobank recruitment date (UK Biobank Field id 130708). ****p < .0001 vs men prescribed simvastatin. △△△△ *p* < .0001 vs women prescribed simvastatin

In order to control for confounding variables, multivariate analysis was utilized in addition to simple bivariate analysis (Figure 1). We created logistic regression models to determine adjusted odds ratios associated with age, T2DM duration, BMI, blood lipids, HbA1c and c‐peptide and statin prescription (with simvastatin as 1‐class and atorvastatin as 0‐class), as the method is ideal for our dataset..^10^ Adjusted odds ratios (ORs), 95% confidence intervals and significance are reported in Tables 3, 4, 5, 6, 7.

## 3 RESULTS

### 3.1 DiaStrat cohort characteristics

From a total of 500 participants, 476 met all inclusion criteria. The average age of the cohort was 62 ± 11 years, and average duration of diabetes was 12 ± 8 years. Eighty five percent of participants had HbA1c values above 48 mmol/mol [6.5%], with an average HbA1c of 65 mmol/mol [8.1%]. Seventy percent of the cohort were classed as obese. The cohort was predominantly men (63%; *p* < .0001; Table 1). HbA1c values in men and women were comparable. There was a greater proportion of obese men than women (*p* < .05); however, obese women had a significantly higher BMI than obese men (38.9 ± 6 vs 35.3 ± 5, *p* < .0001; Table 1). Blood pressure and lipids were generally well managed in the cohort; however, women had significant elevations in HDL (*p* < .0001), LDL (*p* < .05) and total cholesterol (*p* < .001; Table 1) compared with men. Diabetes drug classes and most frequently prescribed non‐diabetes drugs for the DiaStrat Cohort are outlined in Table 2. Biguanides were the most common diabetes drug class (*n* = 375, 73%), and statins were the most common non‐diabetes prescription. Atorvastatin was the most frequently prescribed non‐diabetes medication (*n* = 273, 54%).

**TABLE 1:** DiaStrat cohort characteristics

DiaStrat cohort characteristics	Complete cohort			Men			Women		
	Total	Mean (SD)	%	Total	Mean (SD)	%	Total	Mean (SD)	%
Number of eligible participants	476		95	299		63****	177		37
Age (years) (<80)	476	62 (11)	100	299	62 (10)	100	177	61	100
Duration of diabetes	382	12 (8)	80	244	12 (7)	82	138	12 (9)	78
HbA1c IFCC mmol/mol	441	65 (17)	93	277	65 (16)	63 (93)	164	66 (18)	37 (93)
DCCT %		8.1 (3.7)			8.1 (3.7)			8.2 (3.8)	
>48 mmol/mol (>6.5%)	377	69 (15)	85	240	68 (14)	87	137	70 (17)	84
		8.5 (3.5)			8.4 (3.4)			8.6 (3.7)	
BMI	348	34 (8)	73	219	33 (6)	63 (73)	129	35 (8)	37 (73)
Healthy 18.5–24.9	26	23 (1)	7	16	24 (1)	7	10	23 (1)	8
Overweight 25–29.9	80	28 (1)	23	34	27 (1)	16	33	28 (1)	26*
Obese > 30	242	37 (7)	70	169	35 (5)	77*	86	39 (6)****	67
BP Systolic	311	132 (14)	65	198	132 (13)	64 (66)	113	133.8 (15)	36 (64)
Diastolic	311	76 (9)	65	198	76.2 (9)	64(66)	113	75.8 (10)	36 (64)
Target < 130/80 mmHg	128	120/71	41	90	121/71	45	38	120/70	34
Diagnosed lipid abnormality	209		44	124		60 (41)	85		40 (48)
HDL (mmol/L)	433	1.1 (0.4)	91%	273	1.1 (0.3)	91	160	1.3 (0.3)****	90
LDL (mmol/L)	430	1.9 (0.8)	90%	270	1.8 (0.7)	90	160	2.0 (0.9)*	90
Total cholesterol (mmol/L)	433	3.8 (1.0)	91%	273	3.7 (0.9)	91	160	4.1 (1.0)***	90
Triglycerides (mmol/L)	310	2.1 (1.1)	65%	202	2.1 (1.1)	65%	108	2.0 (1.0)	35%
Number on insulin	180		38	113		63 (38)	67		37 (38)
C‐Peptide (pg/ml)	367	0.66 (0.67)	77	221	0.68 (0.67)	74	140	0.64 (0.68)	79

Note

DiaStrat represents a cohort of T2DM participants recruited from secondary care clinics in northern Ireland. Total number of values available per variable (total), characteristic mean values ± standard deviation (mean (SD)), and percentage of total (%) are illustrated for the complete cohort, men and women. *p < .05, ***p < .001 and ****p < .0001 compared with men or women.

**TABLE 2:** Treatment summary for diabetes drug classes and most frequently prescribed non‐diabetes drugs in the DiaStrat cohort

A. Drug classes used to treat diabetes within the DiaStrat cohort		
Diabetes drug class	Quantity prescribed	% of cohort (+)
Biguanides	365	73
Sulfonylureas	166	33
Short Insulins	133	27
DDP4 inhibitors	95	19
SGLT2 Inhibitor	94	19
Long Insulins	86	17
GLP‐1 mimetics	81	16
Intermediate insulins	32	6
Thiazolidinediones	17	3
Insulin/GLP‐1 mimetics	8	2
Biguanides/DDP4 inhibitors	2	0
Meglitinides	2	0
Thiazolidinedione/biguanides	2	0
Ultralong Insulins	2	0

B. Most frequently prescribed non‐diabetes drugs within the DiaStrat cohort			
Drug name	Drug class	Quantity prescribed	% of cohort (+)
Atorvastatin	Statin	272	54
Aspirin	Antiplatelet drug	224	45
Omeprazole	Proton pump inhibitor	167	33
Amitriptyline	Antidepressant	154	31
Simvastatin	Statin	117	23
Ramipril	ACEi	112	22
Bisoprolol	Beta blocker	99	20
Bendroflumethiazide	Diuretic	92	18
Perindopril	ACEi	85	17
Doxazocin	Alpha‐adrenoceptor blocker	80	16
Irbesartan	Statin	68	14
Levothyroxine	Thyroid hormone	59	12
Rosuvastatin	Statin	58	12
Salbutamol	Bronchodilator	55	12
Clopidogrel	Antiplatelet drug	46	10
Co‐codamol	Opioid Analgesic	44	9
Lansoprazole	Proton pump inhibitor	40	8
Candesartan	Angiotensin II receptor antagonist	33	7
Ezetimibe	Cholesterol absorption inhibitor	33	7

Note

DiaStrat represents a cohort of T2DM participants recruited from secondary care clinics in northern Ireland. Diabetes drug class, quantity prescribed and percentage of total cohort prescribed each diabetes drug class (A), and; drug name, drug class, quantity prescribed and percentage of total cohort prescribed non‐diabetes drugs (B).

### 3.2 Diagnosis of a lipid abnormality in the DiaStrat cohort, particularly in women, is associated with increased HbA1c

There were 217 participants diagnosed with hyperlipidaemia (Table 3). At a cohort level bivariate analysis revealed, those with a diagnosed lipid abnormality were older (64 ± 9 vs 60 ± 11 years, *p* < .0001), had increased duration of T2DM (15 ± 7 vs 9 ± 7 years, (*p* < .001) and reduced LDL (1.7 ± 0.8 vs 2.0 ± 0.8, *p* < .01) compared with those without a lipid abnormality (Table 3). HbA1c was significantly increased in the presence of a lipid abnormality (68 ± 16 [8.4%] vs 63 ± 17; [7.9%] mmol/mol; *p* < .05, Table 3). Only T2DM duration retained significance in multivariate analyses (OR 1.2 (1.10–1.21).

**TABLE 3:** Diagnosis of a lipid abnormality, particularly in women, is associated with increased HbA1c in the DiaStrat cohort

		Age (SD) (years)	T2DM Duration (SD) (years)	BMI (SD) (Kg/m^2^)	Blood Lipids (SD) (mmol/L)				HbA1c (SD) (IFCC mmol/mol) (DCCT %)	C‐Peptide (SD) (pg/ml)	
					HDL	LDL	Total Cholesterol	Triglycerides			
	Total diagnosed with lipid abnormality *n* = 217	64 (9)	15 (7)	34 (8)	1.1 (0.3)	1.7 (0.8)	3.7 (1.0)	2.0 (1.2)	68 (16) 8.4 (3.6)	0.5 (0.4)	

	Total not diagnosed with lipid abnormality	60 (11)****	9 (7)***	34 (7)	1.1 (4)	2.0 (0.8)**	3.9 (0.9)	1.9 (1.0)	63 (17)*	0.7 (0.8)	
	*n* = 259								7.9 (3.7)		
	Adjusted OR (CI)	1.0 (0.98 – 1.04)	1.2*** (1.10–1.21)	1.0 (0.96–1.04)	0.5 (0.24–1.20)	0.6 (0.34–1.24)	1.5 (0.84–2.53)	0.8 (0.44–1.1)	1.0 (1.00–1.21)	1.5 (0.55–4.65)	
Diagnosed with lipid abnormality	Men *n* = 135	**65 (9)△△△**	**16 (8)△△△**	33 (5)	1.1 (0.3)	**1.6 (0.7)△△△**	**3.6 (0.9)△**	2.4 (1.4)	66 (15) 8.2 (3.7)	0.5 (0.5)	

	Women *n* = 82	63 (10)	**15 (7)△△△**	36 (11)	1.2 (0.3)	1.8 (0.9)	3.9 (1.1)*	2.5 (1.4)	**70 (18)** △△ 8.6 (3.8)	0.5 (0.5)	

	Adjusted OR (CI)	1.0 (0.96–1.04)	1.0 (0.96–1.06)	**0.9** ★ **(0.89–0.98)**	0.2 (0.07–0.66)	1.3 (0.57–2.89)	0.8 (0.40–1.46)	1.0 (0.62 (1.43))	0.9 (0.97–1.00)	1.3 (0.45–3.60)	
Not diagnosed with lipid abnormality	Men *n* = 166	60 (11)** ✦✦	9 (6)*** ✦✦✦★★★	34 (7)	1.1 (0.4)	1.9 (0.8)	3.8 (0.9)	2.1 (1.1)	64 (16) 8.0 (3.6)	0.8 (0.7)	

	Women *n* = 93	60 (12)** ✦✦	9 (10)*** ✦✦✦ ★★★	34 (8)	1.3 (0.4)*** △△ ✦✦✦ ФФ	2.1 (0.8)** ✦✦	4.2 (0.9)** ✦✦✦	1.8 (0.8)	**61 (17)** * ✦★★ 7.7 (3.7)	0.7 (0.8)	

	Adjusted OR (CI)	1.0 (0.99–1.11)	0.9 (0.86–1.03)	0.94 (0.88–1.02)	0.02 Ф (0.004–0.19)	0.2 (0.43–1.14)	2.4 (0.64–9.39)	0.7 (0.53–1.42)	1.0 (0.98–1.04)	1.6 (0.45–5.69)	

Note

Mean values ± standard deviation (Mean (SD)), for age, T2DM duration, BMI, HDL, LDL, total cholesterol, triglycerides, HbA1c and C‐peptide of the total DiaStrat cohort with and without a diagnosed lipid abnormality, and for men and women separately. DiaStrat represents a cohort of T2DM participants recruited from secondary care clinics in northern Ireland. Adjusted OR (CI), represents results from logistic regression including all variables. *p < .05, **p < .01, ***p < .001 and ****p < .0001 vs total cohort diagnosed with lipid abnormality. △*p* < .05, △△*p* < .01 and △△△*p* < .001 vs total cohort not diagnosed with lipid abnormality. ✦✦*p* < .01 and ✦✦✦*p* < .001 vs men diagnosed with lipid abnormality. ★★★*p* < .001 vs women with diagnosed with a lipid abnormality. ФФ*p* < .01 vs men not diagnosed with a lipid abnormality. Significant values are highlighted in bold.

In women with a lipid abnormality, bivariate analysis revealed total cholesterol levels were increased compared with the combined cohort with a lipid abnormality (3.9 ± 1.1 vs 3.7 ± 1.0, *p* < .05) and HbA1c increased compared with the combined cohort without a lipid abnormality (70 ± 18 vs 63 ± 17 mmol/L; *p* < .01). Women without a lipid abnormality had increased HDL compared with the total cohort with (*p* < .001) and without (*p* < .001) a lipid abnormality and men with (*p* < .001) and without *(p* < .01) a lipid abnormality. Similarly, LDL was increased in women with a lipid abnormality compared with the total cohort with a lipid abnormality *(p* < .01) and to diagnosed men (*p* < .01). Total cholesterol was highest (4.2 ± 0.9 mmol/L) in women without a lipid abnormality and significantly increased compared with the total cohort with a lipid abnormality (*p* < .01) and with diagnosed men (*p* < .001). HbA1c was lower in women without a diagnosed lipid abnormality than the combined cohort with a lipid abnormality (*p* < .05) and men (*p* < .05) and women (*p* < .01) with a diagnosed lipid abnormality. In contrast, women with a diagnosed lipid abnormality had the highest recorded HbA1c (70 ± 18 mmol/mol [8.6%]), which was significantly higher than the combined cohort without a lipid abnormality (*p* < .01) and women without a diagnosed lipid abnormality (*p* < .01). Multivariate analysis revealed that only BMI was significantly different between men and women with a diagnosed lipid abnormality (OR 0.9 (0.89–0.98), *p* < .05) whilst HDL retained significance between men and women not diagnosed with a lipid abnormality (OR 0.02 (0.004–0.19), *p* < .05).

### 3.3 Simvastatin is associated with superior lipid and glycaemic control to atorvastatin, specifically in women in DiaStrat, and men and women in the UK Biobank

Fifty‐nine men and 36 women were prescribed simvastatin whilst 170 men and 91 women were prescribed atorvastatin in the DiaStrat cohort. Bivariate analysis revealed women prescribed simvastatin had a higher HDL than men prescribed simvastatin (*p* < .05) or atorvastatin (*p* < .01). Both men and women prescribed simvastatin had lower LDL and total cholesterol than women prescribed atorvastatin (*p* < .05–*p* < .001, Table 4). Atorvastatin‐prescribed women had a higher HDL (1.3 vs 1.1 mmol/L, *p* < .001), LDL (2.1 vs 1.8 mmol/L, *p* < .05) and total cholesterol (3.7 vs 4.2 mmol/L, *p* < .01) than atorvastatin‐prescribed men. Comparing within gender and between drugs, a trend was observed suggesting that simvastatin prescription may have a positive effect on HbA1c in women compared with atorvastatin (62 mmol/mol [7.8%] vs 67 mmol/mol [8.3%], *p* = .059). No such differences in HbA1c were observed in men (Table 4). None of the significance was upheld in multivariate analyses.

**TABLE 4:** Characteristics of men and women from the DiaStrat cohort prescribed simvastatin and atorvastatin

		Age (SD) (years)	Duration (SD) (years)	BMI (SD) (Kg/m^2^)	Blood lipids (SD) (mmol/L)				HbA1c (SD) (IFCC mmol/mol) (DCCT %)	C‐Peptide (SD) (pg/ml)	
					HDL	LDL	Total cholesterol	Triglycerides			
Men	Simvastatin (average dose 35 mg/day) *n* = 59	62 (9)	12 (8)	34 (7)	1.1 (0.4)	**1.6 (0.6) △△△**	**3.5 (0.7) △△△**	1.9 (1.0)	65 (15) 8.1 (3.5)	0.7 (0.7)	

	Atorvastatin (average dose 36 mg/day) *n* = 170	62 (10)	12 (8)	33 (6)	1.1 (0.3)	1.8 (0.7)	3.7 (0.9)	1.9 (1.0)	65 (16) 8.1 (3.6)	0.7 (0.7)	

	Adjusted OR (CI)	0.9 (0.92–1.05)	1.0 (0.97–1.13)	1.0 (0.91–1.08)	0.8 (0.15–4.31)	0.7 (0.19–2.33)	0.9 (0.35–2.5)	1.5 (0.55–2.14)	1.0 (0.97–1.04)	6.6 (1.52–2.88)	
Women	Simvastatin (average dose 35 mg/day)	59 (10)	11 (13)	34 (8)	1.3 (0.4) **✦	**1.6 (0.6) △△**	**3.6 (0.7) △**	2.1 (1.2)	62 (17)	0.5 (0.3)	
	*n* = 36								7.8 (3.7)		
	Atorvastatin (average dose 36 mg/day) *n* = 91	63 (10)	12 (7)	36 (12)	1.3 (0.3)***	2.1 (0.9)*	4.2 (1.0)***	2.0 (1.0)	67 (19) 8.3 (3.9)	0.7 (0.8)	
	Adjusted OR (CI)	0.9 (0.87–10.4)	0.8 (0.68–0.94)	0.9 (0.89–1.09)	0.9 (0.10–8.20)	0.2 (0.28–1.72)	1.7 (0.32–9.07)	0.9 (0.33–5.55)	1.0 (0.96–1.05)	0.1 (0.004–4.02)	

Note

DiaStrat represents a cohort of T2DM participants recruited from secondary care clinics in Northern Ireland. Mean values ± standard deviation (Mean (SD)), for age, T2DM duration, BMI, HDL, LDL, total cholesterol, triglycerides, HbA1c and C‐peptide of men and women prescribed simvastatin or atorvastatin. Adjusted OR (CI), represents results from logistic regression including all variables. ** *p* < .01 and *** *p* < .001 vs men prescribed atorvastatin. △ *p* < .05, △△ *p* < .01 and △△△ *p* < .001 vs women prescribed atorvastatin. ✦ *p* < .05 vs men prescribed simvastatin. Significant values are highlighted in bold.

Follow‐up analysis was conducted within the UK Biobank by extracting individuals with T2DM prescribed simvastatin and atorvastatin; 3534 men and 1962 women were prescribed simvastatin and 1431 men and 796 women were prescribed atorvastatin (Table 5). Multivariate analyses revealed that in both men (OR 0.88 [0.79–0.96], *p* < .01) and women (OR 0.84 [0.72–0.98], *p* < .05), triglycerides were significantly reduced in those prescribed simvastatin compared with those prescribed atorvastatin.

**TABLE 5:** Characteristics of men and women with T2DM from the UK Biobank prescribed simvastatin and atorvastatin

		Age (SD) (years)	Duration (SD) (years)	BMI (SD) (Kg/m^2^)	Blood lipids (SD) (mmol/L)				HbA1c (SD) (IFCC mmol/mol) (DCCT %)	
					HDL	LDL	Total cholesterol	Triglycerides		
Men (With T2DM)	Simvastatin *n* = 3534	61 (6)	**5** (4) **	31 (5) **	1.1 (0.3) ****	2.4 (0.6) **	4.0 (0.8) **	2.1 (1.2) ****	54 (14) **	

	Atorvastatin *n* = 1431	61 (6)	6 (5)	32 (5)	1.1 (0.3)	2.4 (0.6)	4.1 (0.9)	2.3 (1.4)	55 (15)	

	Adjusted OR (95% CI)	1.0 (0.98–1.01)	0.98 ** (0.96–0.99)	1.0 (0.98–1.01)	1.2 (0.68–2.12)	0.61 (0.33–1.15)	1.32 (0.78–2.23)	0.88 ** (0.80–0.97)	1.0 (0.99–1.01)	
Women (With T2DM)	Simvastatin *n* = 1962	**60 (7) △**	**5 (5) △**	33 (7)	1.3 (0.3)	**2.6 (0.6) △△**	**4.4 (0.8) △**	**2.0 (1.1) △△**	**54 (14) △**	
	Atorvastatin *n* = 796	61 (6)	6 (4)	33 (7)	1.3 (0.3)	2.6 (0.7)	**4.5 (0.9)**	**2.2 (1.2)**	**56 (15)**	

	Adjusted OR (95% CI)	**0.98 △ (0.97–0.99)**	**0.98 △ (0.95–0.99)**	1.0 (0.98–1.01)	0.71 (0.3–1.71)	0.50 (0.18–1.4)	1.58 (0.67–3.74)	**0.84 △ (0.72–0.98)**	1.0 (0.99–1.00)	

Note

Participant extraction was done using a confirmed diagnosis of T2DM (ICD‐10 code E11, field id 41270). * *p* < .05, ** *p* < .01, **** *p* < .0001 vs men prescribed atorvastatin. △ *p* < .05, △△△△ *p* < .001 vs women prescribed atorvastatin. Significant values are highlighted in bold.

### 3.4 Simvastatin is associated with reduced HbA1c in UK Biobank participants without diabetes

In men without a diabetes diagnosis, multivariate analyses revealed that there was no difference in HDL, LDL or total cholesterol between those prescribed simvastatin and atorvastatin; however, triglycerides (*p* < .05) and HbA1c (*p* < .0001) were reduced in men prescribed simvastatin compared with atorvastatin (Table 6). In women, there was also a significant difference in LDL (*p* < .01), total cholesterol (*p* < .05), triglycerides (*p* < .01) and HbA1c (*p* < .0001), associated with simvastatin compared with atorvastatin, as illustrated in Table 6.

**TABLE 6:** Baseline characteristics of men and women without T1DM or T2DM from the UK Biobank prescribed simvastatin and atorvastatin

		Age (SD) (years)	Duration (SD) (years)	BMI (SD) (Kg/m^2^)	Blood Lipids (SD) (mmol/L)				HbA1c (SD) (IFCC mmol/mol) (DCCT %)	
					HDL	LDL	Total cholesterol	Triglycerides		
Men	Simvastatin *n* = 25593	61 (6) *	‐	29 (4)	1.2 (0.3) ****	2.7 (0.7)	4.6 (0.9)	1.9 (1.0) ****	37 (6) ****	
	Atorvastatin *n* = 6310	61 (6)	‐	29 (4)	1.2 (0.3)	2.7 (0.7)	4.6 (0.9)	2.0 (1.2)	38 (8)	
	Adjusted OR (95% CI)	1.0 (0.99–1.01)		1.01 (0.99–1.02)	1.16 (0.88–1.52)	0.95 (0.69–1.29)	1.06 (0.82–1.38)	0.94* (0.89–0.99)	0.98**** (0.98–0.99)	
Women	Simvastatin *n* = 17223	**62 (6) △△△△**	‐	29 (5)	**1.5 (0.4) △**	**2.9 (0.7) △**	5.0 (0.9)	**1.7 (0.9) △△**	**37 (5) △△△△**	
	Atorvastatin *n* = 3931	62 (6)	‐	29 (5)	1.5 (0.4)	2.9 (0.7)	5.0 (0.9)	1.8 (1.0)	38 (5)	
	Adjusted OR (95% CI)	**0.99 △△△△ (0.98–0.99)**		0.99 (0.98–1.00)	0.72 (0.52–1.01)	**0.58 △△ (0.39–0.86)**	**1.51△ (1.08–2.10)**	**0.90 △△ (0.84–0.97)**	**0.981 △△△△ (0.974–0.989)**	

Note

Prescription information was extracted from data obtained at the initial recruitment appointment for the UK Biobank. All participants with a confirmed diagnosis of T1DM or T2DM (ICD‐10 code E11, field id 41270) were excluded. * *p* < .05, ** *p* < .01, **** *p* < .0001 vs men prescribed atorvastatin. △ *p* < .05, △△ *p* < .01, △△△△ *p* < .0001 vs women prescribed atorvastatin. Adjusted OR (CI), represents results from logistic regression including all variables. Significant values are highlighted in bold.

### 3.5 Simvastatin is associated with fewer incident T2DM cases than atorvastatin in UK Biobank participants

Baseline characteristics of individuals prescribed simvastatin and atorvastatin who developed T2DM after initial recruitment (blood sample collection) to the UK Biobank, are illustrated in Table 7. Multivariate analyses revealed that HbA1c was only significantly reduced in women prescribed simvastatin, without T2DM at baseline (*p* < .05).

**TABLE 7:** Characteristics of men and women prescribed simvastatin and atorvastatin with incident T2DM in the UK Biobank

		Age (SD) (years)	Time to T2DM (SD) (years)	BMI (SD) (Kg/m^2^)	Blood Lipids (SD) (mmol/L)				HbA1c (SD) (IFCC mmol/mol) (DCCT %)	
					HDL	LDL	Total Cholesterol	Triglycerides		
Men	Simvastatin *n* = 5454	61 (7)	6 (3)	31 (5) *	**1.1 (0.3)** **	2.6 (0.7) **	4.3 (0.9) **	2.2 (1.2) ****	47 (12)	
	Atorvastatin *n* = 1617	61 (7)	6 (3)	31 (5)	1.1 (0.3)	2.6 (0.6)	4.4 (0.9)	2.4 (1.4)	48 (12)	
	Adjusted OR (95% CI)	1.0 (0.99–1.01)	1.01 (0.99–1.03)	0.99 (0.98–1.01)	0.88 (0.52–1.47)	0.53 * (0.30–0.93)	1.55 (0.07–2.48)	0.85 **** (0.78–0.92)	1.0 (0.99–1.01)	
Women	Simvastatin *n* = 3080	61 (6)	6 (3)	32 (6)	**1.3 (0.3) △**	**2.7 (0.7) △△**	**4.7 (0.9) △**	2.1 (1.0) *	48 (12)	
	Atorvastatin *n* = 899	61 (6)	6 (3)	32 (6)	1.3 (0.3)	2.8 (0.7)	4.7 (0.9)	2.2 (1.1)	49 (12)	
	Adjusted OR (95% CI)	0.99 (0.98–1.00)	1.01 (0.98–1.03)	1.01 (0.99–1.02)	0.94 (0.44–1.97)	0.46 (0.20–1.05)	1.67 (0.82–3.40)	0.93 (0.80–1.07)	**0.99 △ (0.98–0.99)**	

Note

Incident diabetes was determined when first occurrence of T2DM was recorded after initial UK Biobank recruitment date (UK Biobank Field id 130708). * *p* < .05, ** *p* < .01, **** *p* < .0001 vs men prescribed atorvastatin. △ *p* < .05, △△△△ *p* < .001 vs women prescribed atorvastatin. Adjusted OR (CI), represents results from logistic regression including all variables. Significant values are highlighted in bold.

Approximately 18% of men without T2DM at baseline who were prescribed simvastatin developed T2DM, whereas ~20% prescribed atorvastatin developed T2DM (*p* < .0001, OR 0.83 (0.78–0.88) Table 8). Furthermore, ~15% of women without T2DM at baseline prescribed simvastatin, and ~19% of women prescribed atorvastatin, developed T2DM (*p* < .0001, OR 0.78 (0.72–0.85), Table 8, Figure 1). Furthermore, the odds ratio associated with incident T2DM in men was significantly greater than for women for both simvastatin OR 1.19 (1.14–1.25, *p* < .0001) and atorvastatin OR 1.12 (1.02–1.23, *p* < .05).

**TABLE 8:** Proportion of men and women, without T2DM at baseline, prescribed either simvastatin or atorvastatin that developed T2DM in follow‐up

Incident T2DM in men prescribed simvastatin and atorvastatin within the UK Biobank cohort				
Men	Incident T2DM (iT2DM)	No recorded T2DM in follow‐up	Total	Ratio = iT2DM/Total
Simvastatin	5454	25593	31047	18% *
Atorvastatin	1617	6310	7927	20%

Incident T2DM in women prescribed simvastatin and atorvastatin within the UK Biobank cohort				
Women	Incident T2DM (iT2DM)	No recorded T2DM in follow‐up	Total	Ratio = iT2D/Total
Simvastatin	3080	17223	20303	**15% △**
Atorvastatin	899	3931	4830	19%

Note

Individuals prescribed simvastatin and atorvastatin, without a diagnosis of T2DM, were identified at baseline within the UK Biobank cohort. Incident T2DM was noted when an individual received a T2DM diagnosis after the UK Biobank recruitment date (UK Biobank Field id 130708). * *p* < .0001 vs men prescribed atorvastatin. △ *p* < .0001 vs women prescribed atorvastatin. Significant values are highlighted in bold.

## 4 DISCUSSION

Within the DiaStrat cohort and UK Biobank, we investigated how simvastatin and atorvastatin impacted lipid and glycaemic response and if response differed between men and women. Women responded better to simvastatin in the DiaStrat cohort, but within the UK Biobank, men and women responded better to simvastatin than atorvastatin. Simvastatin also reduced the risk of developing T2DM in both men and women.

In 2008, rosuvastatin was linked to increased diabetes risk in the JUPITER study, and the link between statin treatment and glycaemic control has been of concern since.^10^ It is established that high HbA1c is correlated with elevated lipids.^11^ This often translates into patients being prescribed high‐intensity statins, such as atorvastatin.^12^ Studies have linked statin therapy to diabetes onset and have highlighted high‐dose therapy and LDL level as the main factors influencing diabetes incidence.^13^ The CARDS study found that atorvastatin negatively affected HbA1c in participants with diabetes, but found no effect in a simvastatin‐treated group.^13^ Consistently, we show that both atorvastatin and simvastatin reduce LDL and total cholesterol in men and women, but simvastatin was associated with lower HbA1c than atorvastatin. The effect of statins on glycaemic control is controversial with prior reports of beneficial effects,^14^ negative effects^4^ or no impact at all.^15^ The mechanisms behind the differential effects of statins are not well understood, particularly in relation to the opposing effects of atorvastatin and simvastatin on glucose metabolism.^16^ Most studies show atorvastatin reduces LDL and total cholesterol levels without influencing blood glucose in individuals with diabetes.^17^ The effect of simvastatin appears to be dependent on dose or the cohort profile. In those with T2DM and hypercholesterolemia, simvastatin doses of 80 mg/day result in a ~10% increase in plasma glucose after 2 months,^18^ whilst lower dose (<20 mg/day) statins have less impact on glycaemia.^19^ Other work has reported that 20 mg/day simvastatin negatively affects insulin sensitivity but has no effect on insulin or glucose levels after 4 weeks.^20^ These conflicting findings highlight complexities relating to simvastatin dose and duration of prescription. These complexities are also evident in people with T2DM and no specified hypercholesterolemia, where Szendroedi et al.,^21^ showed 80 mg/day simvastatin treatment had no effect on insulin sensitivity, fasting insulin levels or HOMA‐B levels. Hydrie and colleagues^22^ reported people with T2DM and insulin resistance showed improved glycaemic control after receiving 40mg/day simvastatin for 3 months, which is consistent with our observations. Our study also supports the VYTAL^23^ study's finding that simvastatin has greater lipid‐lowering effects than atorvastatin and the VOYAGER^24^ study's observation that simvastatin causes a superior (2.1% greater) reduction in LDL in women than men.

We utilized the UK Biobank to assess if the sex specific effect of simvastatin on lipid control and HbA1c was evident in the wider UK population with a T2DM diagnosis. In the UK Biobank analysis, simvastatin was associated with superior lipid control in both men and women. The differences observed may be attributed to dosing inconsistencies.^12^ In the DiaStrat cohort, both simvastatin and atorvastatin were prescribed at an average dose of ~40 mg/day, which is considered high‐intensity therapy.^12^ The standard dose of atorvastatin is 10 mg/day^25^ and simvastatin 20 mg/day^26^ and it is likely that a wider range of dosage regimens were represented within the UK Biobank cohort. Our findings suggest that in DiaStrat, high‐intensity atorvastatin therapy negatively impacts lipid and HbA1c control in women, whilst in the wider UK Biobank population, representative of low or moderate dosing, simvastatin is universally more effective at lowering LDL, cholesterol and HbA1c. There is now compelling evidence showing statin dose has variable effects dependant on ethnicity and comorbidity profile,^27^ which may be of significance within the UK Biobank analysis, associated with larger population variation.

In the UK Biobank, those without diabetes prescribed simvastatin had a lower HbA1c than those prescribed atorvastatin, and had fewer cases of incident T2DM. This is of high clinical relevance and supported by the VYTAL study, which showed simvastatin to be superior than atorvastatin in participants with T2DM for lipid control and suggests potential benefits for diabetes progression.^23^ A large multicentre trial recruited participants with metabolic syndrome, including people with diabetes, and showed simvastatin caused significant reductions in LDL, consistently greater increases in HDL and greater reductions in metabolic syndrome criteria compared with atorvastatin.^28^

It is widely accepted that statins increase the risk of T2DM. In the women's health initiative study, moderate statin therapy had a significant effect on diabetes risk with a Hazard Ratio (HR) of 1.5.^29^ In another UK study, with 2 million participants followed over 15 years, statin‐associated risk was significant (HR 3.6), with no difference between moderate or intensive therapy.^30^ Other work has shown high‐intensity statins such as atorvastatin and rosuvastatin are associated with higher risk than moderate intensity statins, such as simvastatin.^19^ These findings correspond with the present study which reports reduced incidence of T2DM in the UK Biobank with simvastatin but not atorvastatin, and a higher risk in men compared with women when exposed to either drug.

This analysis has several limitations. DiaStrat recruitment was from one geographical region, and the cohort may represent a severe T2DM phenotype due to the fact that recruitment was conducted in a secondary care setting. This likely contributed to an atorvastatin prescription bias when compared with simvastatin. Patients >80 years where excluded from the DiaStrat study, and this cohort represents a large number of statin prescriptions.^31^ Future studies should not impose an upper age limit, given the relevance of older adults to the topic. This was also the case in the UK Biobank, which excludes those >70 years. This limitation may be addressed in future longitudinal studies as the UK Biobank participants age. The high rate of CVD, obesity and related CVD risk evident in this cohort commonly results in patients starting on intensive therapy.^12^ The generalizability of findings, however, was substantiated by follow‐up analyses within the UK Biobank, representing a significantly larger cohort. Prescription data within the UK Biobank were obtained via verbal interview, which undoubtedly impacts reliability. Consideration needs to be made that prescription guidelines for DiaStrat would have differed from UK Biobank. Historically, simvastatin would have been prescribed at a higher dose, over atorvastatin. Prior to 2010, the Joint British Societies' guidelines (JBS 2) aimed for LDL of <2 mmol/L in high‐risk individuals rather than the current 40% reduction in non‐HDL cholesterol.^32^ Furthermore, comorbidities and associated polypharmacy may play a role in our observed findings. There are few longitudinal studies looking at the effect of individual statins and further prospective studies are warranted. A recent longitudinal study (11 years), in non‐diabetic patients, has reported that atorvastatin and simvastatin increased the fasting blood glucose.^33^ Given the potential importance of reducing incident T2DM and improving glycaemic control in established T2DM, outcomes reported here should be investigated in randomized controlled trials. The present study cannot definitively establish that simvastatin and atorvastatin are responsible for differences in lipids and HbA1c observed, rather that they are associated with this observation. We aimed to address confounding but utilizing a multivariate analysis approach; well‐designed prospective studies will determine the reproducibility of our observations and potentially identify other unmeasured confounders.

## 5 CONCLUSION

In the DiaStrat cohort, simvastatin and atorvastatin were associated with reduced LDL and total cholesterol in T2DM participants, whilst simvastatin was associated with superior glycaemic control in women. In the UK Biobank, superiority of simvastatin over atorvastatin, in terms of glycaemic and lipid control, was observed in both men and women. Furthermore, in individuals without T2DM at baseline, atorvastatin is associated with increased risk of incident T2DM when compared with simvastatin. Whilst causality cannot be established within the present study, our observations suggest that simvastatin is associated with superior lipid‐lowering and HbA1c properties in those at‐risk of, and diagnosed with, T2DM and may, after confirmatory clinical trials, inform prescribing practices in this population.

## CONFLICT OF INTEREST

The authors have no conflicts of interests to declare.

## AUTHOR CONTRIBUTION

**Andrew R. English:** Data curation (lead); Formal analysis (lead); Investigation (lead); Visualization (lead); Writing – original draft (lead). **Bodhayan Prasad:** Data curation (supporting); Formal analysis (supporting); Methodology (supporting); Software (lead); Writing – review and editing (supporting). **Declan H. McGuigan:** Data curation (supporting); Formal analysis (supporting); Investigation (supporting); Writing – review and editing (supporting). **Geraldine Horigan:** Data curation (supporting); Writing – review and editing (supporting). **Maurice OKane:** Data curation (supporting); Project administration (supporting); Writing – review and editing (supporting). **Anthony J. Bjourson:** Conceptualization (lead); Formal analysis (supporting); Investigation (supporting); Writing – review and editing (supporting). **Priyank Shukla:** Data curation (supporting); Formal analysis (supporting); Investigation (supporting); Methodology (supporting); Supervision (supporting); Writing – review and editing (supporting). **Catriona Kelly:** Conceptualization (lead); Formal analysis (supporting); Investigation (supporting); Project administration (lead); Supervision (lead); Writing – review and editing (supporting). **Paula L. McClean:** Conceptualization (lead); Formal analysis (supporting); Investigation (supporting); Project administration (lead); Supervision (lead); Writing – review and editing (supporting).

## Data Availability

The data that support the findings of this study are available on request from the corresponding author. The data are not publicly available due to privacy or ethical restrictions.
